# Supplementary material for: Automatic Text-Mining Approach to Identify Molecular Target Candidates Associated with Metabolic Processes for Myotonic Dystrophy Type 1
Source: Int J Environ Res Public Health. 2023 Jan 27;20(3):2283. doi: 10.3390/ijerph20032283 (PMC9915907; doi:10.3390/ijerph20032283)
Supplement: Supplementary file 1 [file ijerph-20-02283-s001.zip › Supplementary Figures_Identifying novel metabolic process targets for Myotonic Dystrophy type 1.pdf]

# Supporting Information

## Identifying novel metabolic process targets for Myotonic Dystrophy type 1

Dhvani H Kuntawala[1] Filipa, Martins[1] Rui Vitorino[1] Sandra Rebelo[1*]

[1]Medical Science Department, Institute of Biomedicine – iBiMED, University of Aveiro, 3810-183 Aveiro, Portugal

Corresponding author: srebelo@ua.pt

Sandra Rebelo, Principal Investigator
Institute of Biomedicine – iBiMED
Department of Medical Sciences, University of Aveiro 3810-183 Aveiro, Portugal
Email: srebelo@ua.pt

| | |
|---|---|
| **Supplementary Figure 1** | Network map for VOSviewer. |
| **Supplementary Figure 2** | Protein-protein interactions (PPI) from STRING database. |

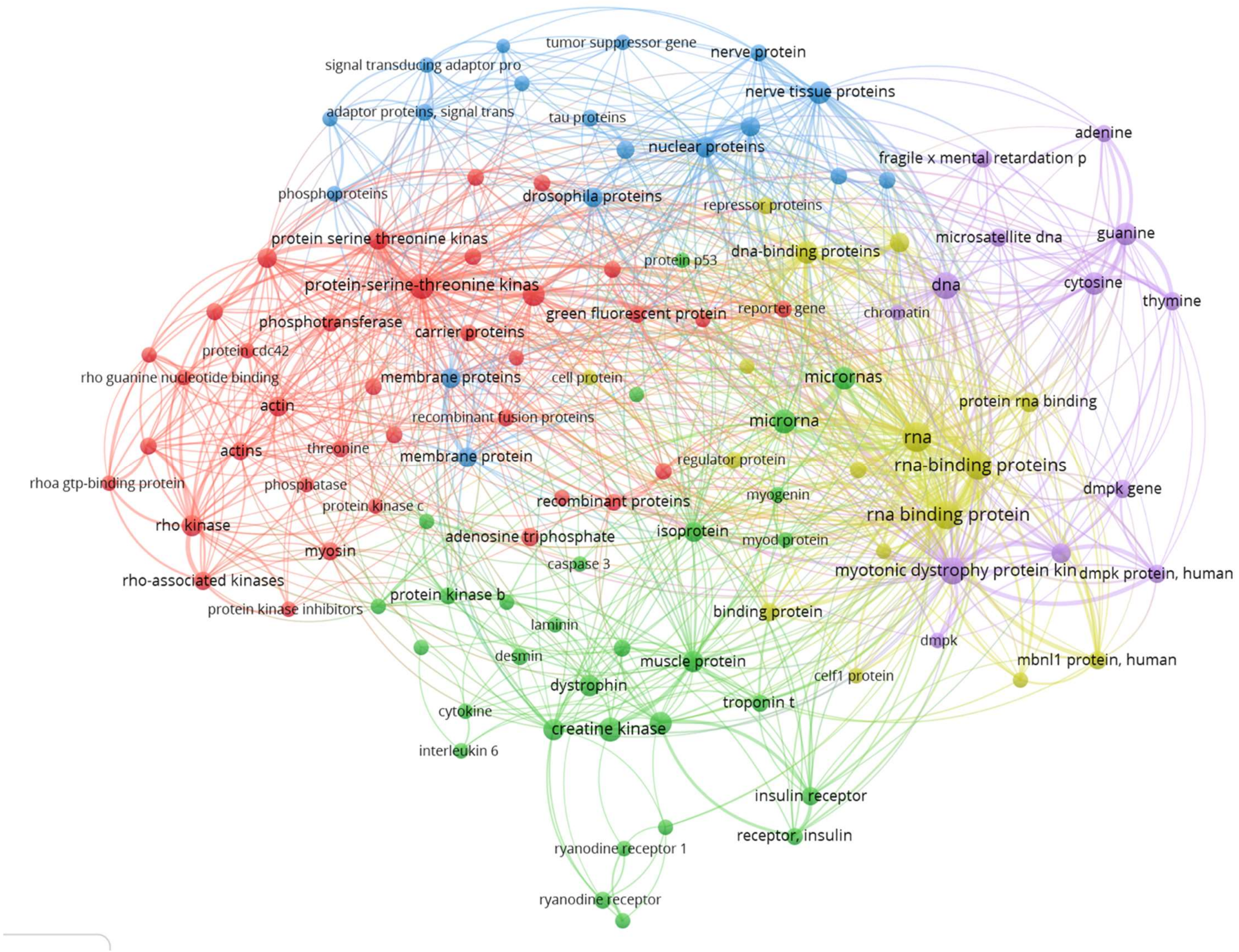


**Figure S1 | Network map.** Occurrence distribution of the similar genes found both in Scopus and DisGeNET. Different colors are seen by the terms divided into clusters. Cluster 1 (Red), Cluster 2 (Green), Cluster 3 (Blue), Cluster 4 (Yellow), and Cluster 5 (Purple) represent five clusters.

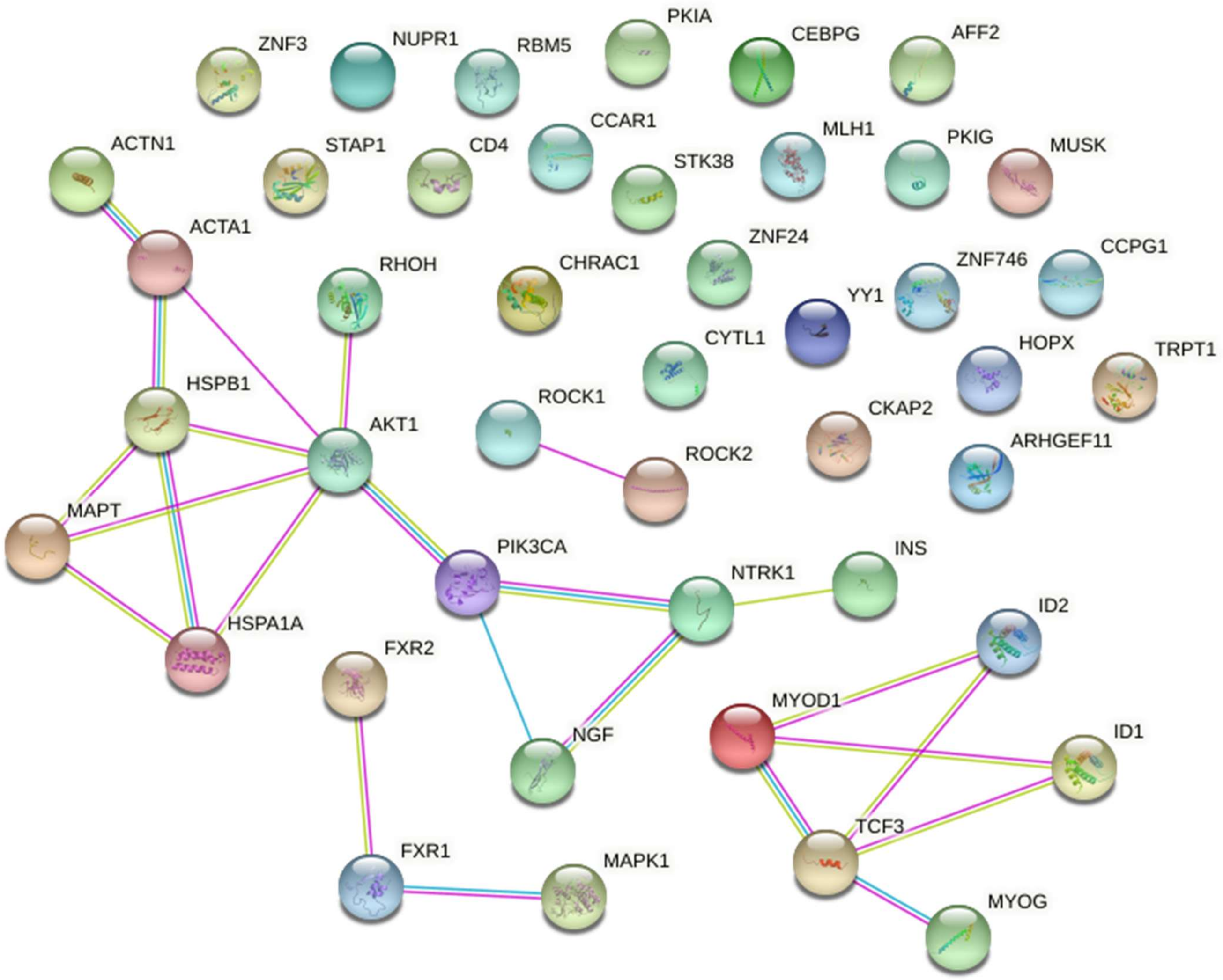


**Figure S2 - PPI network comprising the 44 genes identified and associated with GO biological processes related to protein metabolism**. The network was constructed using the STRING database by applying the physical subnetwork type. The edges indicate that the directly linked proteins are part of the same physical complex, although in large complexes this may not signify, they are directly binding to each other. The edge color denotes the interaction sources: blue edges denote known interactions from curated databases, pink edges denote known interactions experimentally determined, and green edges denotes interactions inferred by text mining.
